# CXCR5-negative natural killer cells ameliorate experimental autoimmune myasthenia gravis by suppressing follicular helper T cells

**DOI:** 10.1186/s12974-019-1687-x

**Published:** 2019-12-29

**Authors:** Chun-Lin Yang, Peng Zhang, Ru-Tao Liu, Na Zhang, Min Zhang, Heng Li, Tong Du, Xiao-Li Li, Ying-Chun Dou, Rui-Sheng Duan

**Affiliations:** 1 Department of Neurology, Shandong Provincial Qianfoshan Hospital, Shandong University, No. 16766, Jingshi Road, Jinan, 250014 People’s Republic of China; 2 Department of Neurology, The First Affiliated Hospital of Shandong First Medical University, Jinan, 250014 People’s Republic of China; 3 College of Basic Medical Sciences, Shandong University of Traditional Chinese Medicine, Jinan, 250355 People’s Republic of China

**Keywords:** Experimental autoimmune myasthenia gravis, Natural killer cells, C-X-C chemokine receptor 5 (CXCR5), Follicular helper T cells

## Abstract

**Background:**

Recent studies have demonstrated that natural killer (NK) cells can modulate other immune components and are involved in the development or progression of several autoimmune diseases. However, the roles and mechanisms of NK cells in regulating experimental autoimmune myasthenia gravis (EAMG) remained to be illustrated.

**Methods:**

To address the function of NK cells in experimental autoimmune myasthenia gravis in vivo, EAMG rats were adoptively transferred with splenic NK cells. The serum antibodies, and splenic follicular helper T (Tfh) cells and germinal center B cells were determined by ELISA and flow cytometry. The roles of NK cells in regulating Tfh cells were further verified in vitro by co-culturing splenocytes or isolated T cells with NK cells. Moreover, the phenotype, localization, and function differences between different NK cell subtypes were determined by flow cytometry, immunofluorescence, and ex vivo co-culturation.

**Results:**

In this study, we found that adoptive transfer of NK cells ameliorated EAMG symptoms by suppressing Tfh cells and germinal center B cells. Ex vivo studies indicated NK cells inhibited CD4^+^ T cells and Tfh cells by inducing the apoptosis of T cells. More importantly, NK cells could be divided into CXCR5^-^ and CXCR5^+^ NK subtypes according to the expression of CXCR5 molecular. Compared with CXCR5^-^ NK cells, which were mainly localized outside B cell zone, CXCR5^+^ NK were concentrated in the B cell zone and exhibited higher expression levels of IL-17 and ICOS, and lower expression level of CD27. Ex vivo studies indicated it was CXCR5^-^ NK cells not CXCR5^+^ NK cells that suppressed CD4^+^ T cells and Tfh cells. Further analysis revealed that, compared with CXCR5^-^ NK cells, CXCR5^+^ NK cells enhanced the ICOS expression of Tfh cells.

**Conclusions:**

These findings highlight the different roles of CXCR5^-^ NK cells and CXCR5^+^ NK cells. It was CXCR5^-^ NK cells but not CXCR5^+^ NK cells that suppressed Tfh cells and inhibited the autoimmune response in EAMG models.

## Introduction

Myasthenia gravis (MG) is an antibody-mediated autoimmune disease which is mainly caused by antibodies targeting acetylcholine receptor (AChR) at the postsynaptic neuromuscular junction membrane, leading to impaired signal transduction, muscle weakness, and fatigability [1]. Other MG-specific autoantibodies include antibodies against muscle-specific kinase and lipoprotein receptor-related protein 4 [2].

Follicular helper T (Tfh) cells are the major CD4^+^ T cell subset involved in humoral immune response. Tfh cells, which are characterized by the expression of CXCR5, can migrate into B cell follicles, and promote the proliferation, differentiation, and antibody class-switching of B cells. Tfh cells express high levels of inducible co-stimulator (ICOS), programmed death 1 (PD-1), and IL-21, all of which are essential for antibody production and generation memory B cells with high-affinity antibodies. Exaggerated Tfh cell responses have been demonstrated in a broad range of autoimmune diseases such as systemic lupus erythematosus and autoimmune thyroid diseases [3]. Altered circulating Tfh cells or Tfh cell subtypes in patients with myasthenia gravis have also been reported in several recent literatures [4, 5]. It was also demonstrated that intra-thymic Tfh cells could interact with B cells, leading to ectopic germinal centers formation and anti-AChR antibody production [6]. Reduction of Tfh cells could ameliorate the symptom of experimental autoimmune myasthenia gravis (EAMG) in mice [7]. All of these studies suggest a pathogenic role of Tfh in MG/EAMG.

Natural killer (NK) cells are large granular lymphocytes with the capacities to directly recognize and kill foreign, infected, and malignant cells [8]. Growing evidences from mouse and human studies demonstrate that NK cells can also modulate other aspects of the immune system and are involved in the development or progression of the autoimmune diseases, through their production of numerous cytokines and chemokines or by perforin-mediated cell death [8–10].

NK cells limited autoimmune occurrence by controlling CD4^+^ T cell and CD8^+^ T cell responses in the TNF-related apoptosis-inducing ligand (TRAIL) or perforin-dependent manner [11–13]. It has been shown that NK cells isolated from patients with MS had decreased frequency and effector function [10]. Transfer of acetylcholine-producing NK cells into the cerebral ventricles of experimental autoimmune encephalomyelitis (EAE) mice attenuated brain and spinal cord damage via modulation of infiltrating monocytes and macrophages [14]. Recent studies also demonstrated an inhibitory role of NK cells in regulation of Tfh cells in lymphocytic choriomeningitis virus model and NP-KLH (4-hydroxy-3-nitrophenylacetyl hapten conjugated to Keyhole Limpet Hemocyanin) immunization model [15, 16]. However, it is unclear whether NK cells could modulate Tfh cells and their subtypes in EAMG model and other autoimmune diseases.

## Materials and methods

### Animals

Female Lewis rats (6–8 weeks) were purchased from Vital River Laboratories (Beijing, China), kept under specific pathogen-free conditions and 12/12 light-dark schedule, and provided with standard pellet diet and water ad libitum. Animals were killed by isoflurane inhalation and decapitation. All the experimental protocols were approved by the institutional ethics committee.

### Induction of EAMG

Rat AChR97–116 peptide (DGDFAIVKFTKVLLDYTGHI), corresponding to rat AChR alpha subunit, were purchased from China Peptides Co. Ltd (Shanghai, China). Rat EAMG models were induced as previously reported [17]. In brief, the rats were immunized subcutaneously at the tail base with a total 200 μl emulsion containing 75 μg of rat AChR97–116 peptide in phosphate buffer saline (PBS) emulsified in an equal volume of complete Freund’s adjuvant containing 1 mg mycobacterium tuberculosis (strain H37RA; Difco, Detroit, MI, USA). Then, the rats were boosted on day 30 with the same dose of AChR97–116 in incomplete Freund’s adjuvant. After the first immunization, body weights and clinical scores were assessed every other day in the blinded manner by two researchers at the same time as follows: 0, normal strength and no abnormalities; 1, mildly decreased activity and weak grip or cry, more evident at the end of exercise; 2, clinical signs present before exercise (tremor, head down, hunched posture, weak grip); 3, severe clinical signs present before exercise, no grip, moribund; and 4, dead.

### Induction of experimental autoimmune neuritis (EAN)

Bovine peripheral myelin (BPM) was isolated from fresh adult bovine cauda equina as previously reported [18]. To induce EAN rat model, ten female Lewis rats, aged 6–8 weeks, were subcutaneously immunized with 200 μl emulsion containing 1 mg BPM in saline and an equal volume of complete Freund’s adjuvant containing 0.3 mg Mycobacterium tuberculosis (strain H37RA; Difco, Detroit, MI, USA) at the tail base. At the day 13 post-immunization (p.i.), the rats were sacrificed and the spleens were obtained for flow cytometry analysis.

### Flow cytometry and intracellular cytokine staining

Spleens or lymph nodes were minced through the 70-μm cell strainers to generate single-cell suspensions. The red blood cells of splenic single-cell suspensions were lysed by RBC lysis buffer (Biolegend) for 5 min on ice. The cells were stained according to standard protocols with the following antibodies: anti-rat CD25 (OX39; Invitrogen), CD161 (10/78; BD Pharmingen), CD80 (3H5; eBioscience), CD86 (24F; Biolegend), CD4 (OX35; eBioscience), MHC-II (OX-6; Biolegend), Foxp3 (FJK-16S; eBioscience), IL-17A (eBio17B7; eBioscience), CD3 (1F4; Biolegend), CD103 (OX62; Biolegend), IFN-γ (DB1; Invitrogen), ICOS (C398.4A; Biolegend), B220 (HIS24; eBioscience), CXCR5 (EPR8837; Abcam), and peanut agglutinin (PNA) (Vector Laboratories). Unconjugated primary antibodies were detected with Alexa Fluor 488-conjugated anti-rabbit IgG (Abcam). The following monoclonal antibodies were used for mouse cell staining: CD4 (GK1.5; eBioscience), NK1.1 (PK136; Biolegend), PD-1 (29F.1A12; Biolegend), CD3 (145-2C11; eBioscience), CXCR5 (2G8; BD Pharmingen), CXCR5 (L138D7; Biolegend), and CD19 (eBio1D3; eBioscience).

For intracellular cytokine staining, cells were stimulated for 5 h at 37 °C with cell stimulation cocktail (eBioscience). Stimulated cells were then pre-incubated with PBS containing 0.5% bovine serum albumin and stained for 30 min at 4 °C with various combinations of fluorescently tagged antibodies. After washing, cells were fixed in 2% paraformaldehyde and then permeabilized using intracellular fixation and permeabilization buffer set (eBioscience, 88-8824-00) and then stained with antibodies specific for various cytokines. Flow cytometry was performed on BD FACS Aria II (BD Biosciences).

### Isolation of NK cells by magnetic beads and FACS

The isolation of NK cells by magnetic cell sorting was performed in two steps according to the manufacturer’s instructions. Briefly, splenocytes were stained with Alexa Fluor 647-conjugated anti-rat CD3 (Biolegend) mAb and PE-conjugated anti-rat CD161 (BD Biosciences) mAb. Then, the cells were incubated with anti-Alexa Fluor 647 microbeads (Miltenyi Biotec). Subsequently, the cell suspension was loaded on a selection column placed in a magnetic field to deplete the CD3-positive cells. Secondly, the flow-through fraction was incubated with anti-PE microbeads (Miltenyi Biotec). Finally, the cell suspension was loaded on a selection column placed in a magnetic field and the magnetically retained fraction was the NK cell fraction. The purity of NK cells, as analyzed by flow cytometry, was routinely > 90%.

Isolation of NK cells for ex vivo culture was performed by sorting CD3^-^CD161^bright^ or CD3^-^NK1.1^+^ cells from rat or mouse spleen using a BD FACS Aria II. The purities were about 95%. To isolate CXCR5^+^ and CXCR5^-^ NK cells, the NK1.1- or CD161-positive cells were enriched from mouse or rat splenocytes by magnetic cell sorting. Then, CXCR5^+^ and CXCR5^-^ NK cells were sorted from NK1.1 or CD161 positive fraction by BD FACS Aria II with the purity about 85%. Isolation of T cells for ex vivo culture was performed by sorting CD3^+^ T cells from rat or mouse spleen using a BD FACS Aria II with the purity about 95%.

### NK cell adoptive transfer

5 × 10^6^ magnetically sorted NK cells in 300 μl PBS were injected intravenously (i.v.) into recipient EAMG rats twice at the day before the first and second immunization, respectively. The control rats received the same volumes of PBS at the corresponding time.

To test the migration of NK cells in vivo, magnetically sorted NK cells or BD FACS Aria II sorted CXCR5^+^ and CXCR5^-^ NK cells were labeled for 15 min at 37 °C with the 2 μM fluorescent dye carboxyfluorescein diacetate, succinimidyl ester (CFSE) (CFDA-SE, Molecular Probes), washed, and transferred i.v. (5 × 10^6^ cells for NK cells or 0.5 × 10^6^ cells for CXCR5^+^ and CXCR5^-^ NK cells) to recipient immunized rats. Two days later, the spleens were harvested and applied for immunofluorescent stain.

### ELISA testing for antibody levels and affinities

Rat serum anti-AChR97–116 IgG, IgG1, IgG2a, and IgG2b antibody levels were detected by ELISA as described previously [17]. The following reagents were used: biotin rabbit anti-rat IgG (Poly4054; BioLegend), IgG1 (MRG1-58; BioLegend), IgG2a (MRG2a-83; BioLegend), IgG2b (MRG2b-85; BioLegend), streptavidin-horseradish peroxidase (Biosynthesis Biotechnology), and tetramethylbenzidine (TMB) substrate (Tiangen Biotechnology, Beijing, China). The serum anti-AChR97–116 IgG antibody affinities were determined by the thiocyanate method as described previously [19]. OD values were read at a wavelength of 450 nm by using a microplate ELISA reader.

### Immunofluorescence microscopy

Cryosections of 8 μm were fixed in cold acetone and stained with mouse anti-rat CD161 (10/78; BD Pharmingen) and fluorescein-conjugated PNA (Vector Laboratories) or rabbit anti-rat CXCR5 (Abcam), followed by PE-conjugated rabbit anti-mouse IgG or Alexa Fluor 488-conjugated anti-rabbit IgG (Abcam). For NK cell tracing experiment, cryosections were fixed and stained with Dylight 594-conjugated anti-rat IgM (Abcam). Images were captured with fluorescence microscopy (Olympus FSX100, Tokyo, Japan).

### Cell culture

Immunized rat or mouse splenocytes were re-suspended to 1–2 × 10^6^/ml in RPMI 1640 (containing 2.05 mM glutamine; HyClone, Beijing, China) supplemented with 1% (v/v) penicillin–streptomycin (containing 10,000 IU/ml penicillin and 10,000 μg/ml streptomycin; HyClone, Logan, UT, USA) and 10% (v/v) fetal bovine serum (FBS; Biological Industries, Israel) for the following experiments. (i) Rat splenocytes were co-cultured with NK cells or not (splenocytes: NK = 5: 1) in the presence of IL-2 for three days. (ii) Mouse splenocytes or T cells were co-cultured with NK cells or not (splenocytes: NK = 5: 1) in the presence of 1 μg/ml anti-CD3, 1 μg/ml anti-CD28, and 10 ng/ml IL-15 for 3 days. (iii) Splenocytes or T cells were co-cultured with CXCR5^-^ NK cells, CXCR5^+^ NK cells, or not (splenocytes or T: NK = 5: 1) in the presence of anti-CD3, anti-CD28, and IL-15 for 3 days. (iv) Mouse splenocytes were cultured with IL-15 or not in the presence of anti-CD3 and anti-CD28 for 3 days. (v) Mouse splenocytes were labeled with CFSE and cultured with IL-15 or not in the presence of lipopolysaccharide (LPS) for three days. Then, the cells were harvested, stained with corresponding antibodies and detected by using a flow cytometry.

### Statistical analysis

Results are routinely displayed as mean ± SEM, with statistical differences between experimental groups determined using a two-tailed unpaired Student’s *t* test, one-way ANOVA, and Spearman correlation test, where a *p* value of < 0.05 was deemed significant. Graphs were produced, and statistical analyses were performed using GraphPad Prism.

## Results

### NK cells ameliorate EAMG symptoms and reduce serum anti-AChR97-116 antibody levels and antibody affinities

**Fig 1:**
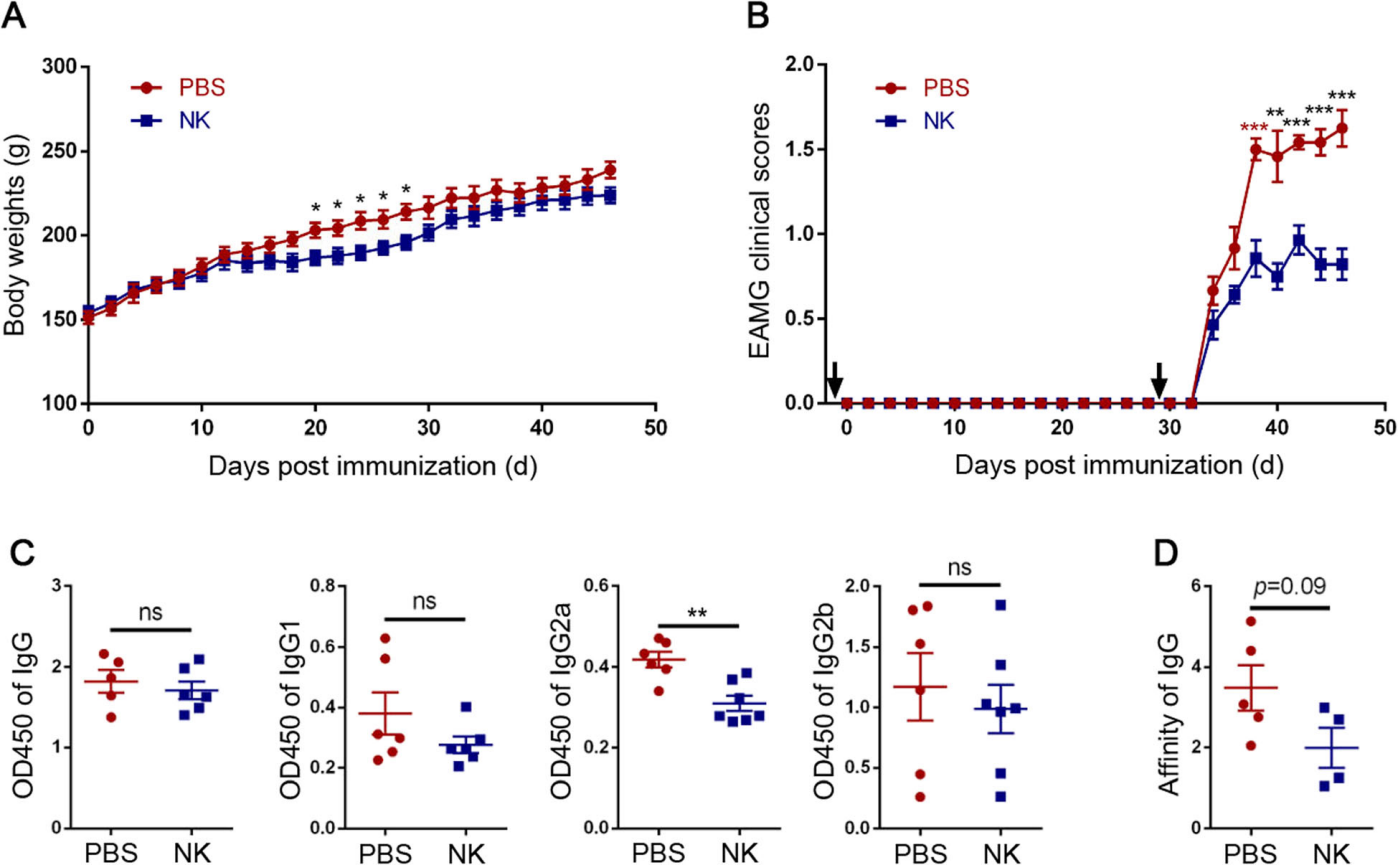
NK cell ameliorated EAMG symptoms and reduced serum anti-AChR97–116 IgG2a antibodies levels. NK cells were adoptively transferred into EAMG rats twice at the day before the first and second immunization, respectively. The body weights (**a**) and clinical scores (**b**) of NK cell-treated rats (*n* = 7) and PBS-treated rats (*n* = 6) were recorded every other day after the first immunization. The rats were sacrificed at the 46th day post the first immunization, and the blood sera were collected. Serum anti-AChR97–116 IgG, IgG1, IgG2a, and IgG2b antibody levels were determined by ELISA (**c**). Anti-AChR97–116 IgG antibody affinity was as determined by the thiocyanate method (**d**). Data were presented as mean ± SEM. Results were representatives of two independent experiments. Unpaired Student’s *t* test was used. Arrows mean intervention times. *p < 0.05, *p < 0.01, ***p < 0.001

To test for the regulatory roles of NK cells in EAMG, splenic NK cells (5 × 10^6^) from donor rats were isolated and transferred into recipient EAMG rats twice at the day before the first and second immunization, respectively. Compared with control rats, NK cell-treated rats had lower clinical scores (Fig. 1b), associated with reductions of anti-AChR97–116 IgG2a antibody levels (Fig. 1c). There was a trending but not statistically significant decrease of anti-AChR97–116 IgG antibody affinities in NK cell-treated group (*p* = 0.09, Fig. 1d). However, we did not find any differences in the concentrations of anti-AChR97–116 IgG, IgG1, or IgG2b between those two groups (Fig. 1c). Interestingly, transient body weight loss from days 20 to 28 post-immunization (p.i.) was observed in NK cell-treated group (Fig. 1a).

### NK cell adoptive transfer reduces Tfh and germinal center B cells in EAMG

**Fig. 2:**
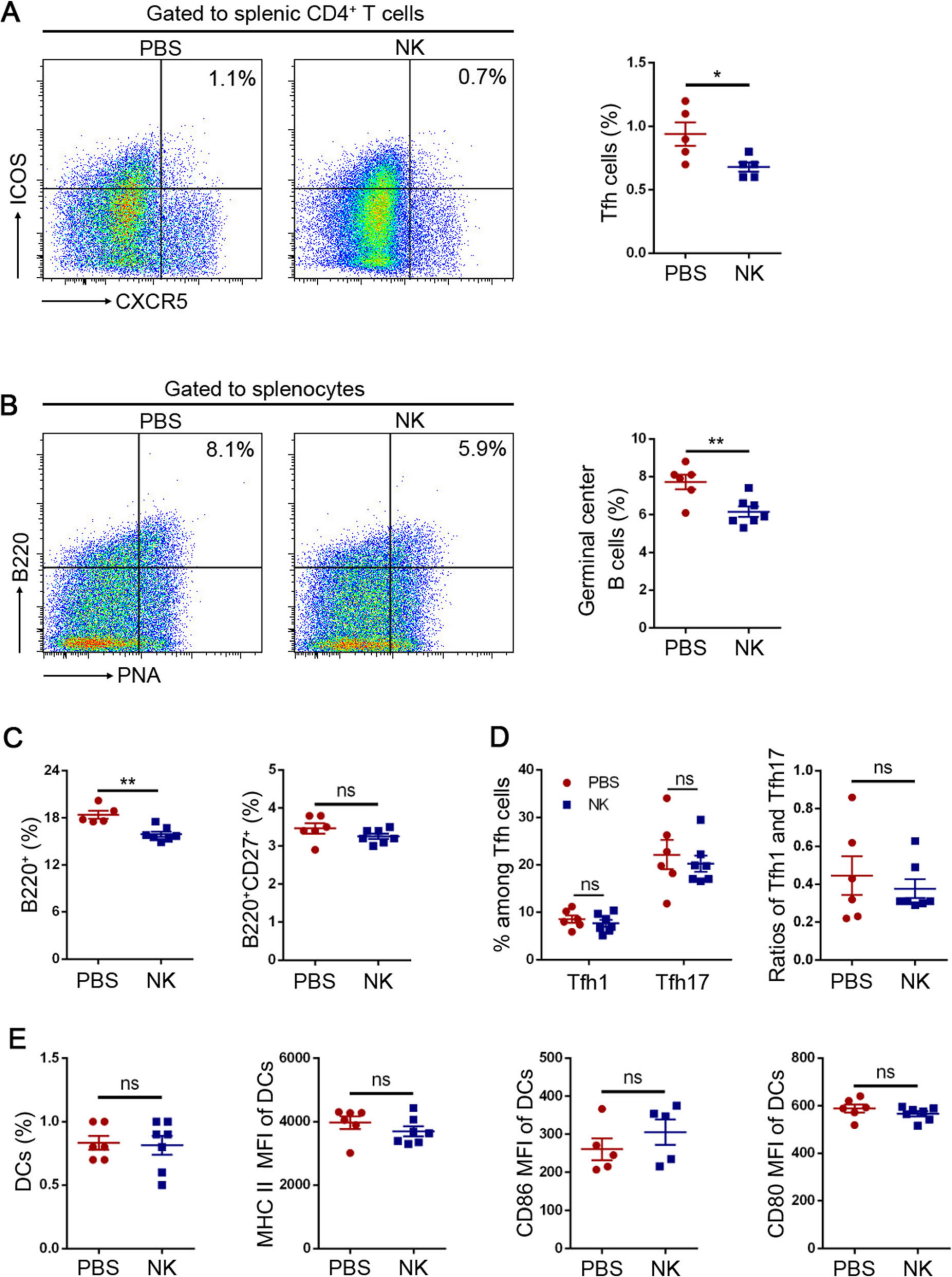
NK cell adoptive transfer reduced Tfh cells and germinal center B cells in EAMG. Rats were sacrificed at the 46th day post the first immunization, and the spleens were collected. The following results were representatives of two independent experiments. **a** Frequencies of Tfh cells (CD4^+^CXCR5^+^ICOS^+^) among splenic CD4^+^ cells were downregulated by NK cell treatment. **b** Frequencies of germinal center B cells (B220^+^PNA^+^) among splenocytes were downregulated after NK cell adoptive transfer. **c** B cell (B220^+^) frequencies but not memory B cell (B220^+^CD27^+^) frequencies among splenocytes were reduced after NK cell adoptive transfer. **d** NK cell adoptive transfer did not change IFN-γ^+^ (Tfh1) or IL-17^+^ (Tfh17) cell frequencies among Tfh cells (CD4^+^CXCR5^+^ICOS^+^). **e** Frequencies and phenotypes of splenic DCs after NK cell treatment in EAMG. Dendritic cells were gated as CD103^+^MHC II^+^. The phenotypes of DCs referred to CD80, CD86, and MHC II expression levels, which were shown as MFI (mean fluorescent intensity). Data were presented as mean ± SEM. Unpaired Student’s *t* test was used. ns means not significant, *p < 0.05, **p < 0.01

Considering that recent reports illustrated regulatory functions of NK cells in humoral immune response [15, 16], we decided to further examine the potential roles of NK cells in the regulation of Tfh cells and germinal center B cells. Consistently, EAMG rats treated with NK cells exhibited lower percentages of Tfh cells and germinal center B cells compared to untreated rats (Fig. 2a, b). A slight but statistically significant decrease of B cell percentages in the NK cell-treated group was also observed (Fig. 2c, left). However, the percentages of memory B cells were not changed by NK cell treatment (Fig. 2c, right). The previous study illustrated Tfh cells with different cytokine profiles could modulate the affinity and isotype of the antibody response. Tfh1 cells, characterized by IFN-γ production, were essential for IgG2a class switching [20]. Because adoptive transfer of NK cells led to decreased anti-AChR97–116 IgG2a antibody levels, we further determined whether NK cells could modulate Tfh cells subtypes, which include Tfh1 and Tfh17 cells. However, our results revealed that NK cell adoptive transfer regulated the percentage of neither Tfh1 cells nor Tfh17 cells (Fig. 2d, left). Also, the ratios of Tfh1 to Tfh17 were not different between the NK cell-treated and the control rats (Fig. 2d, right).

Dendritic cells (DCs) are the most potent professional antigen-presenting cells which play pivotal roles in driving Tfh cell differentiation [21–23]. To elucidate the mechanism of NK cells to regulate humoral immune response, we figure out whether NK cells could modulate the number or function of DCs. However, we did not observe either the number or phenotype changes of DCs between those two groups (Fig. 2e).

### NK cells inhibit CD4^+^ T cells and Tfh cells by increased apoptosis of T cells and Tfh cells in vitro

**Fig 3:**
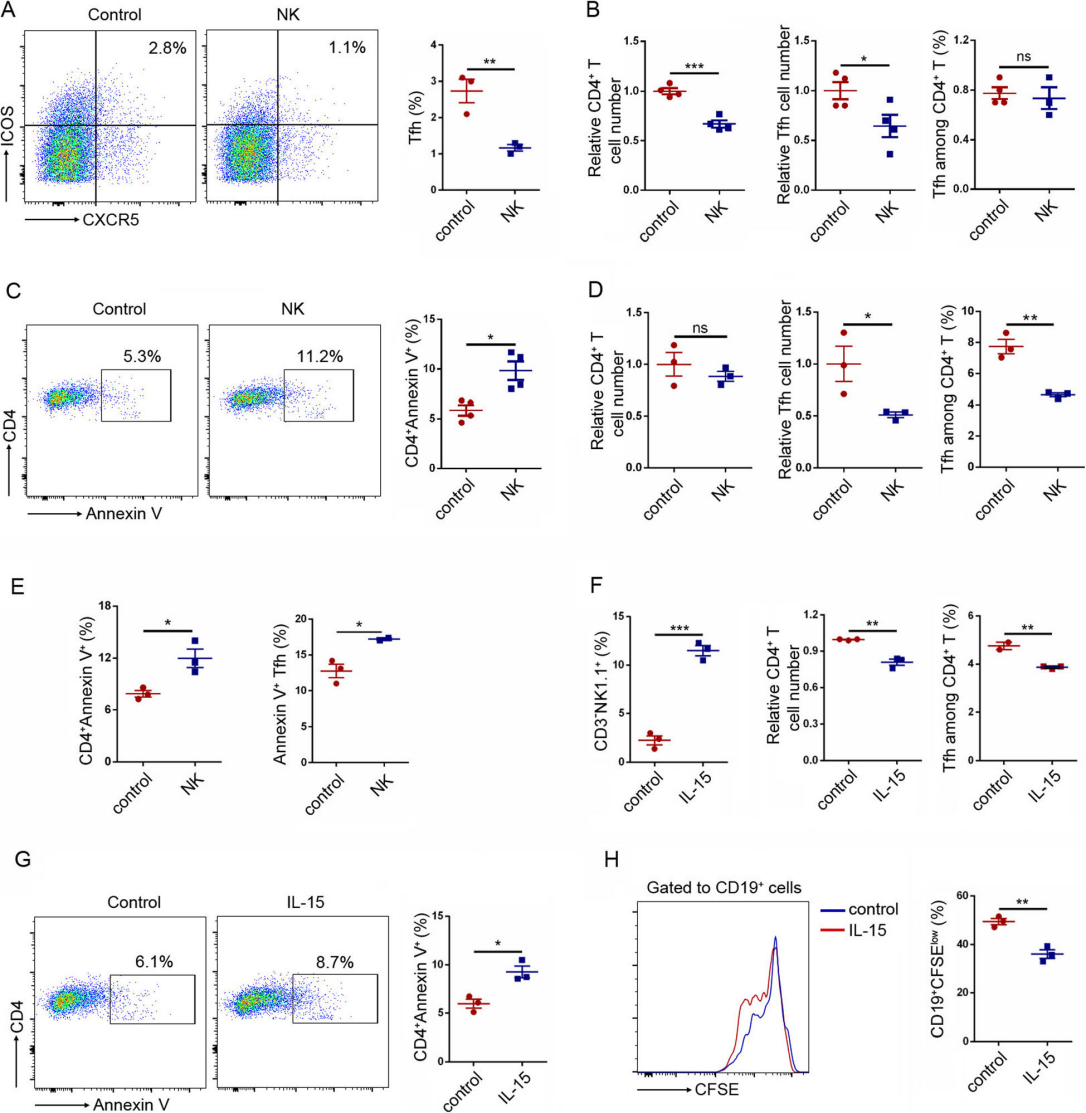
NK cells inhibit CD4^+^ T cells and Tfh cells in vitro. **a** Rat splenocytes were co-cultured with NK cells or not. Representative flow cytometric plots of Tfh cells (CD4^+^CXCR5^+^ICOS^+^) among CD4^+^ T cells (left). Frequencies of Tfh cells in CD4^+^ T cells were downregulated by NK cells. **b**, **c** Mouse splenocytes were co-cultured with NK cells or not. CD4^+^ T cell and Tfh (CD4^+^CXCR5^+^PD1^+^) cell numbers were counted by flow cytometry and shown in relative forms (**b**, left and median). The Tfh cell frequencies in CD4^+^ T cells were not changed (**b**, right). The apoptosis of CD4^+^ T cells was determined by staining cells with CD4 and Annexin V (**c**). **d**, **e** Splenic CD3^+^ T were co-cultured with NK cells or not. The CD4^+^ T cells and Tfh cells were determined by flow cytometry (**d**). The apoptosis of CD4^+^ T cells and Tfh cells was determined by staining cells with Annexin V (**e**). **f**, **g** Mouse splenocytes were cultured with IL-15 or not. IL-15 increased NK cells dramatically in vitro (**f**, left). IL-15 treatment decreased CD4^+^ T cell numbers and Tfh cell frequencies in CD4^+^ T cells (**f**, median and right) and induced apoptosis of CD4^+^ T cells (**g**). **h** Mouse splenocytes were labeled with CFSE and cultured with IL-15 or not. Cells were then stained with CD19 to detect the proliferation of B cells. Representative flow cytometric histogram of CFSE dilutions of B cells. Data were from one (**a**, **h**) or two independent experiments (**b**–**g**). Data were presented as mean ± SEM. Unpaired Student’s *t* test was used. ns means not significant, *p < 0.05, **p < 0.01, ***p < 0.001

Previous work demonstrated that NK cells could eliminate activated CD4^+^ and CD8^+^ T cells, which is mediated by TRAIL and perforin in a contact-dependent manner [11–13]. To address the effects of NK cells on Tfh cells, isolated NK cells were co-cultured with splenocytes for 3 days in vitro. Co-culturing rat splenic cells with NK cells led to a substantial reduction in the percentage of Tfh cells (Fig. 3a). The numbers of CD4^+^ T cells and Tfh cells were also decreased when mouse splenic cells were co-cultured with NK cells (Fig. 3b). However, the frequencies of mouse Tfh in CD4^+^ T cells were not changed (Fig. 3b). To investigate the mechanism of NK cells in regulating T cells and Tfh cells, the mouse splenocytes were stained with Annexin V after co-cultured with NK cells and found that NK cells increased apoptosis of CD4^+^ T cells (Fig. 3c).

To exclude the influence of other cell components in splenocytes which might mediate the suppressive effects of NK cells on Tfh cells, splenic CD3^+^ T cells were isolated and co-cultured with NK cells. Consistently, NK cells decreased Tfh cells (Fig. 3d) and induced the apoptosis of CD4^+^ T cells and Tfh cells (Fig. 3e). These results indicated NK cells directly inhibited T cells and Tfh cells by inducing their apoptosis.

### IL-15 increases NK cells and decreases CD4^+^ T cells and Tfh cells in vitro

To further verify the inhibition effects of NK cells on Tfh cells, splenocytes were treated with IL-15, a well-known NK cell stimulatory cytokine [24]. IL-15 could significantly increase NK cell percentages in the cultured splenocytes (Fig. 3f). Consistently, IL-15-treated groups had lower levels of CD4^+^ T cells and Tfh cells compared with control groups by inducing apoptosis of T cells (Fig. 3f, g), which confirmed from one perspective that NK cells could regulate Tfh cells. Additionally, IL-15 inhibited LPS-induced B cell proliferation (Fig. 3g), and this might attribute to its inhibiting effects on CD4^+^ T cells and Tfh cells.

### NK cells can be divided into CXCR5^-^ and CXCR5^+^ NK subtypes

**Fig. 4:**
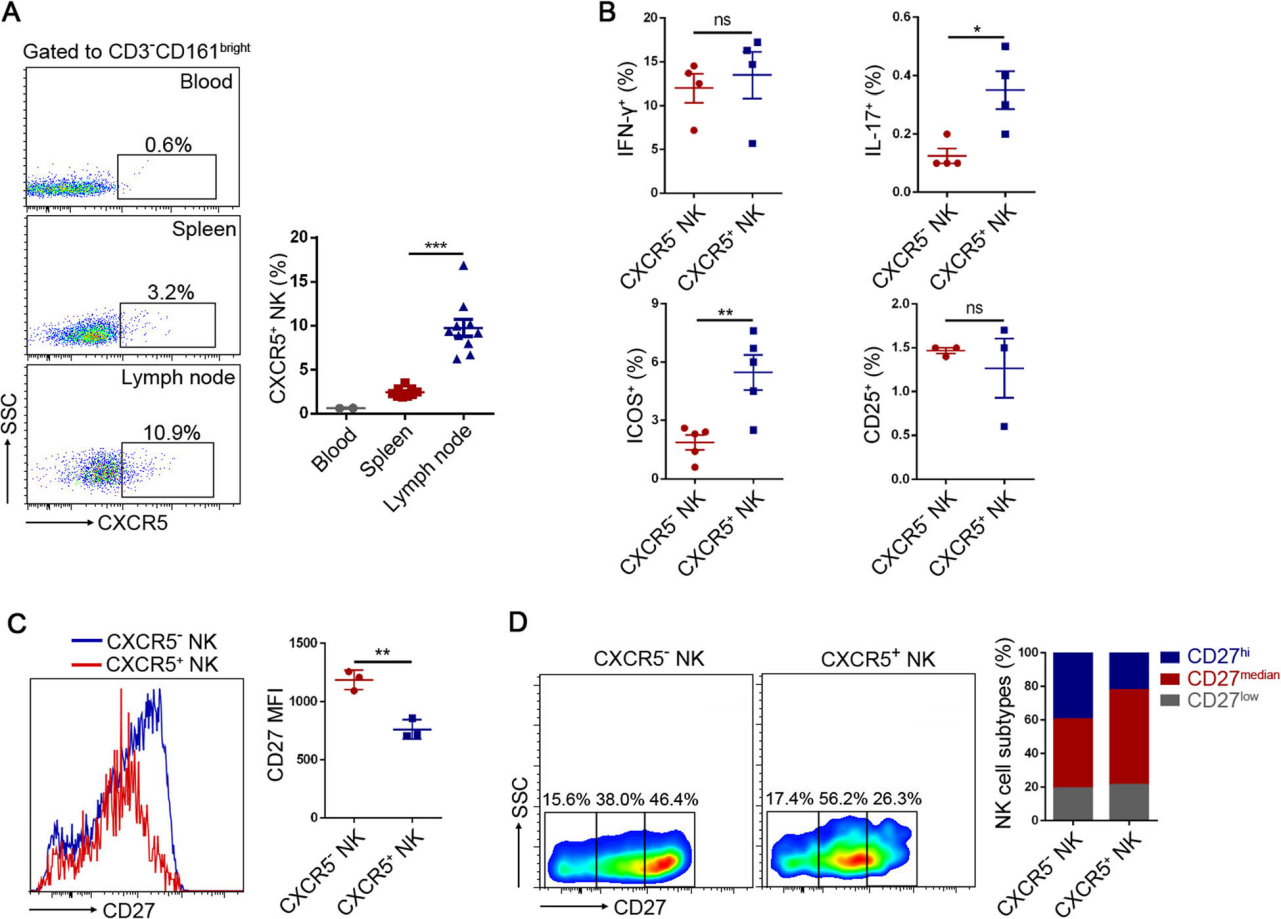
Phenotype differences between CXCR5^-^ and CXCR5^+^ NK cells. **a** Frequencies of CXCR5^+^ cell among blood, lymph node, and spleen CD3^-^CD161^bright^ NK cells. Data were presented as mean ± SEM. One-way ANOVA. **p < 0.01. **b** Frequencies of IFN-γ^+^, IL-17^+^, ICOS^+^, and CD25^+^ cells within rat CXCR5^-^ and CXCR5^+^ NK cells. Data were presented as mean ± SEM. Results were representatives of two or three independent experiments. Unpaired Student’s *t* test was used. ns means not significant, *p < 0.05, **p < 0.01. **c** Representative flow cytometric histogram of the expression of CD27 on rat CXCR5^-^ and CXCR5^+^ NK cells (left). Mean fluorescent intensity (MFI) of CD27 expression of rat CXCR5^-^ and CXCR5^+^ NK cells (right). Data were presented as mean ± SEM. Three independent experiments. Unpaired Student’s *t* test was used. **p < 0.01. **d** Divide rat CXCR5^-^ and CXCR5^+^ NK cells into three subsets (CD27^low^, CD27^median^, and CD27^hi^) according to CD27 expression levels (left). Frequencies of CD27^low^, CD27^median^, and CD27^hi^ cells among CXCR5^-^ and CXCR5^+^ NK cells (right). Three independent experiments. Data were presented as mean

CXCR5 molecular is expressed by B cells and Tfh cells, which directs those cells to localize to B cell follicles. Our results indicated NK cells could also be divided into CXCR5^-^ and CXCR5^+^ NK subtypes according to the expression of CXCR5 molecular. CXCR5^+^ NK cells were indeed shown in periphery blood, spleen, and lymph node and accounted for 0.63 ± 0.03%, 2.44 ± 0.17%, and 9.75 ± 0.96% of NK cells (means ± SEM), respectively (Fig. 4a). CXCR5^+^ NK cells produced higher levels of IL-17 than the CXCR5^-^ subset, although comparable frequencies of IFN-γ-producing and CD25-positive cells were shown in both subsets (Fig. 4b). Furthermore, CXCR5^+^ NK cells exhibited higher expression level of ICOS (Fig. 4b) and lower of CD27 than their CXCR5^-^ counterparts (Fig. 4c). Moreover, according to the expression level of CD27, both CXCR5^-^ and CXCR5^+^ NK cells could be divided into three subsets (CD27^low^, CD27^median^, and CD27^hi^, respectively) (Fig. 4d). Indeed, CXCR5^+^ NK cells had higher frequencies of CD27^median^ cells and lower frequencies of CD27^hi^ cells compared with CXCR5^-^ NK cells, and these two NK cell subtypes had equal percentages of CD27^low^ cells (Fig. 4d). Because CD27 is firstly expressed by immature NK cells and downregulated after maturation, these results indicated CXCR5^+^ NK cells might be more maturated than CXCR5^-^ NK cells.

### Distribution and migration of CXCR5^-^ and CXCR5^+^ NK cells

**Fig. 5:**
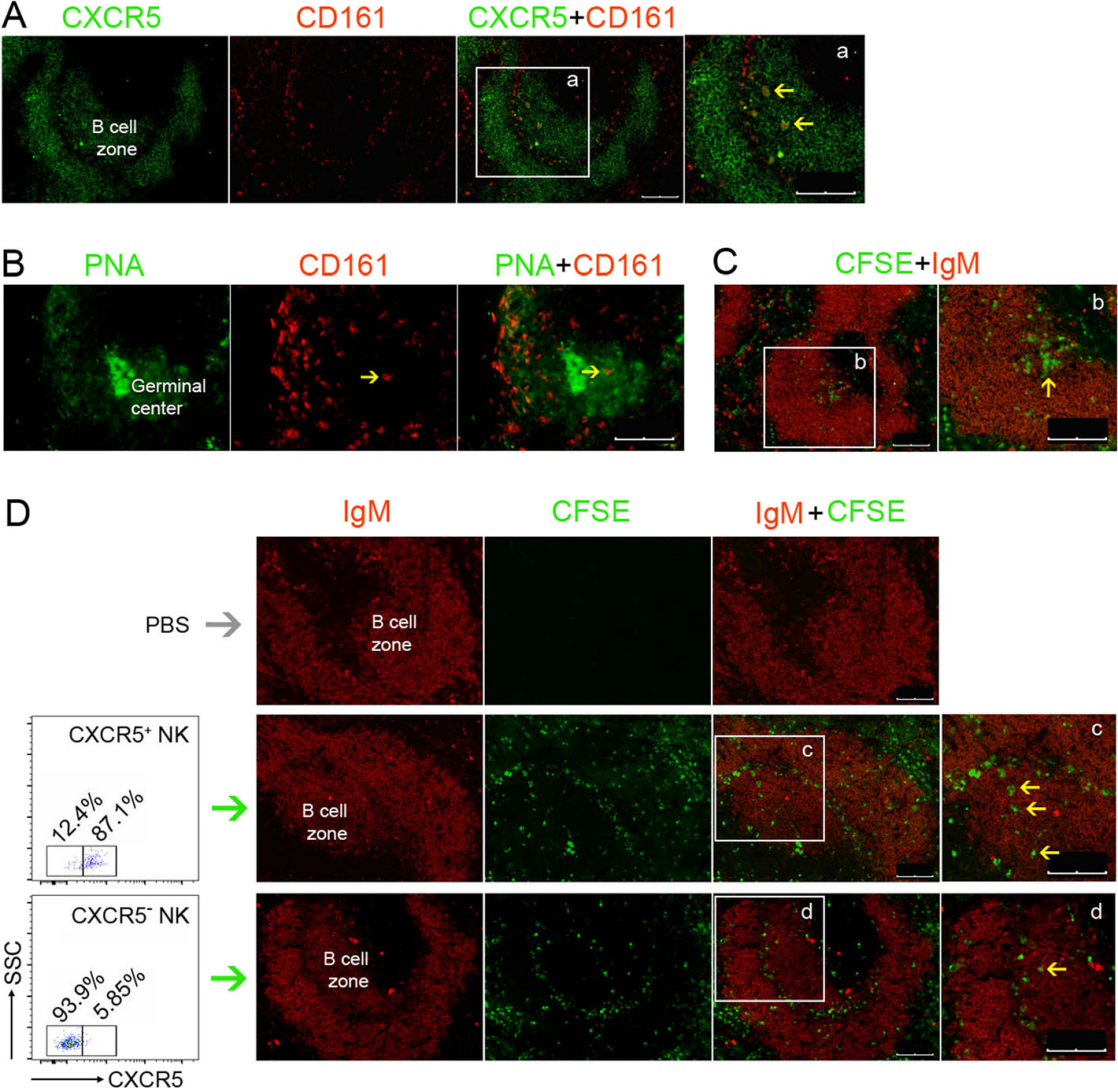
Distribution and migration of CXCR5^+^ and CXCR5^-^ NK cells. **a** Fluorescence image of a spleen from EAMG rat. Red indicated CD161, and green indicated CXCR5 (B cell zone). Arrowhead indicated CD161^+^CXCR5^+^ cells in the B cell zone. Scale bar, 100 μm. **b** Fluorescence image of EAMG rat lymph node. Red indicated CD161, and green (PNA) indicated germinal center area. Arrowhead indicated CD161^+^ cells in the germinal center area. Scale bar, 100 μm. **c** Some of NK cells migrated into B cell zone after adoptive transfer. NK cells, sorted by MACS sorting, were labeled with CFSE (green) and transferred into EAMG rats. Two days later, the spleen was harvested and stained with anti-IgM antibody (red). Arrowhead indicated NK cells in B cell zone, scale bar, 100 μm. **d** FACS sorted CXCR5^+^ and CXCR5^-^ NK cells were labeled with CFSE (green) and transferred into EAMG rats. Two days later, the spleen was harvested and stained with the anti-IgM antibody (red). Arrowhead indicated NK cells in B cell zone, scale bar, 100 μm

To figure out the different functions of CXCR5^-^ and CXCR5^+^ NK cells in regulating humoral immune response, the distribution and migration of CXCR5^-^ and CXCR5^+^ NK cells were investigated. To identify the localization of NK cells by immunofluorescence, CD161 and CXCR5 were stained with the corresponding antibodies. CD161 were used to indicate NK or natural killer T (NKT) cells. The molecular CXCR5 are mainly expressed by B cells; thus, it can be used as the marker for the B cell zone. Immunofluorescent results indicated CD161^+^CXCR5^+^ cells were concentrated in the B cell zone while CD161^+^CXCR5^-^ cells were mainly localized outside the B cell zone (Fig. 5a). This indicated that the CXCR5^+^ NK cells or NKT cells were localized in the B cell zone. Germinal centers are the sites in the B cell zone, which are formed after immune challenges and are essential for the antibody class switch and affinity maturation. To determine whether NK cells were localized in the geminal center, the frozen sections were stained for CD161 and germinal center with anti-CD161 antibody and PNA, respectively. Our results illustrated there were CD161^+^ cells in the lymph node PNA-positive area, suggesting the existence of NK cells or NKT cells in the germinal center (Fig. 5b). To directly assess whether NK cells could migrate into B cell zone, CFSE-labeled splenic NK cells were transferred to the immunized rats. The labeled NK cells were indeed found in the B cell zone of the spleen (Fig. 5c), which indicated some of NK cells could migrate into B cell zone. To figure out the difference of migration abilities between the CXCR5^-^ and CXCR5^+^ NK cells, the CXCR5^-^ and CXCR5^+^ NK cells were sorted, labeled with CFSE, and transferred to different recipients. Results demonstrated that CXCR5^+^ NK cells could more efficiently migrate into B cell zone than the CXCR5^-^ subtype (Fig. 5d). A tiny number of NK cells were also observed in the B cell zone of spleen which received CXCR5^-^ NK cells. This might be attributed to the contaminated CXCR5^+^ NK cells among the purified CXCR5^-^ NK cells. These results indicated CXCR5^-^ and CXCR5^+^ NK cells might regulate immune response in different sites of the secondary lymphoid organs. CXCR5^-^ NK cells might regulate immune response outside the B cell zone, while CXCR5^+^ NK cells might migrate into B cell follicles and regulate humoral immune response in the B cell area.

### Tfh cells and germinal center cells are positively correlated with CXCR5^+^ NK cells

**Fig. 6:**
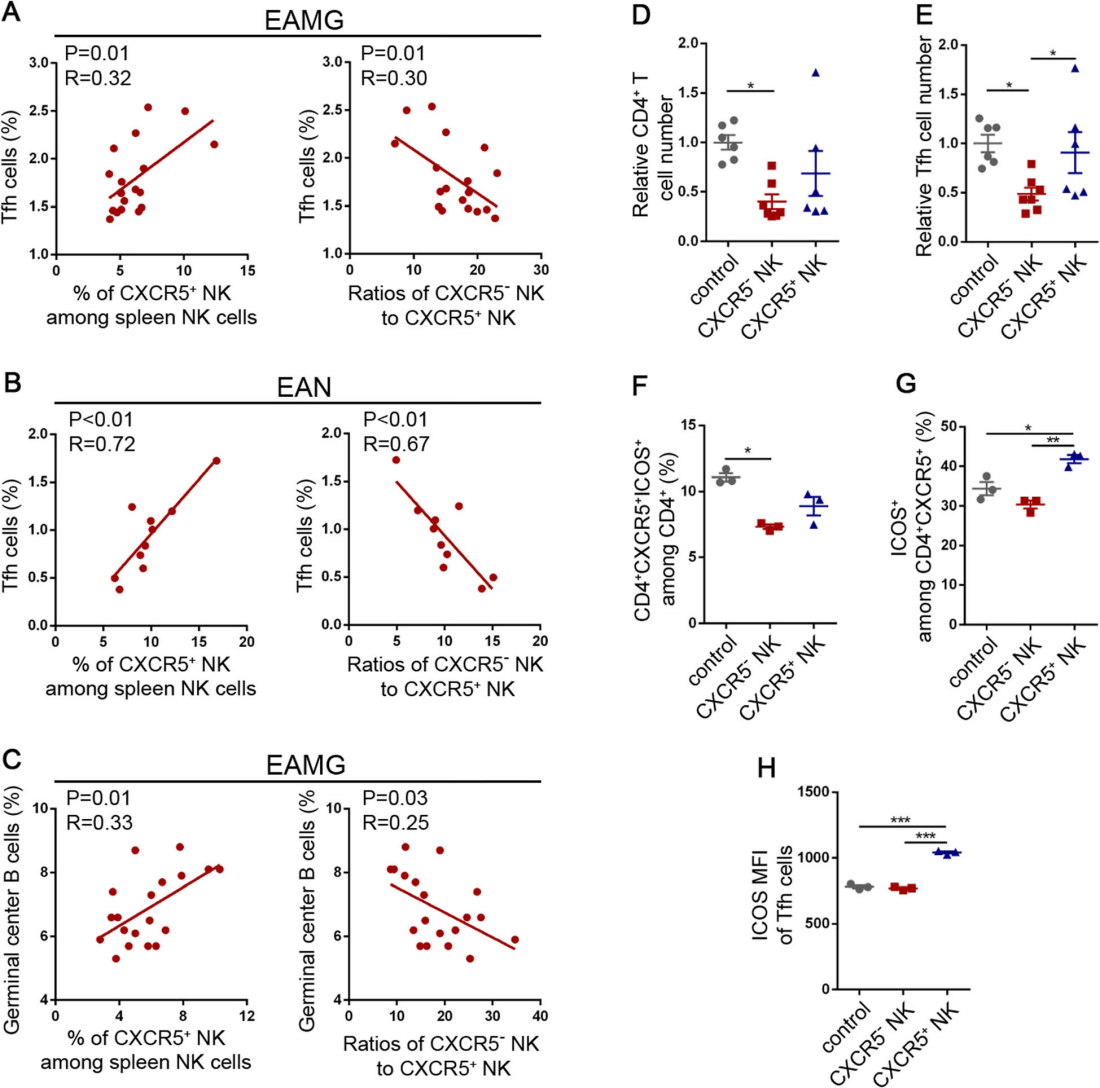
Different roles of CXCR5^-^ and CXCR5^+^ NK cells in regulating Tfh cells. **a**, **b** Correlation analysis of Tfh cell frequencies (among CD4^+^ T cells) with the frequencies of CXCR5^+^ NK cells (left) or the ratios of CXCR5^-^ to CXCR5^+^ NK cells (right) in rat EAMG (**a**) and EAN (**b**) models. Spearman correlation test was used. One-independent experiment. **c** Correlation analysis of germinal center B cell frequencies (among splenocytes) with the frequencies of CXCR5^+^ NK cells (left) or the ratios of CXCR5^-^ to CXCR5^+^ NK cells (right) in rat EAMG. Spearman correlation test was used. One-independent experiment. **d**, **e** Mouse splenocytes were co-cultured with CXCR5^-^ and CXCR5^+^ NK cells. CD4^+^ T cells (**d**) and Tfh (**e**) cell numbers were counted by flow cytometry and shown as relative forms. Data were presented as mean ± SEM. Two independent experiments. One-way ANOVA. *p < 0.05. **f**–**h** Splenic CD3^+^ T were co-cultured with CXCR5^-^ and CXCR5^+^ NK cells for three days and analyzed by flow cytometry. MFI means mean fluorescent intensity. Data were presented as mean ± SEM. Two independent experiments. One-way ANOVA. *p < 0.05, **p < 0.01, ***p < 0.001

To investigate the potential impact of CXCR5^-^ and CXCR5^+^ NK cells on Tfh cells, we firstly evaluated the correlation between Tfh cells and CXCR5^+^ NK cells in rat EAMG models by Spearman correlation test. Interestingly, Tfh cell frequencies had a positive correlation with CXCR5^+^ NK cell frequencies in EAMG rats (Fig. 6a, left). Consistently, Tfh cell frequencies were negatively correlated with the ratios of CXCR5^-^ to CXCR5^+^ NK cells (Fig. 6a, right). Similar results were also observed in experimental autoimmune neuritis (EAN) model rats (Fig. 6b). In addition, the frequencies of germinal center B cells were also positively correlated with CXCR5^+^ NK cell frequencies (Fig. 6c, left) and negatively correlated with the ratios of CXCR5^-^ to CXCR5^+^ NK cells in EAMG rats (Fig. 6c, right). These results indicated that CXCR5^-^ and CXCR5^+^ NK cells might have different roles in regulating Tfh cells or germinal center B cells.

### CXCR5^-^ NK cells but not CXCR5^+^ NK cells suppressed Tfh cells

To directly address the different roles of CXCR5^-^ and CXCR5^+^ NK cells on Tfh cells, mouse splenic CXCR5^+^ NK or CXCR5^-^ NK cells were co-cultured with splenocytes and found that CXCR5^-^ NK cells but not CXCR5^+^ NK and could reduce CD4^+^ T cell and Tfh cell numbers (Fig. 6d, e). Similar results were obtained when isolated splenic CD3^+^ T cells were co-cultured with splenic CXCR5^-^ or CXCR5^+^ NK cells (Fig. 6f). Further analysis illustrated that compared with CXCR5^-^ NK cells, CXCR5^+^ NK cells increased ICOS^+^ cells percentages among CD4^+^CXCR5^+^ T cells (Fig. 6g). Meanwhile, CXCR5^+^ but not CXCR5^-^ NK cells upregulated ICOS mean fluorescent intensity (MFI) of Tfh cells (Fig. 6h). These results illustrated that CXCR5^-^ NK cells and CXCR5^+^ NK cells have different functions in regulate immune response and CXCR5^-^ NK cells could more efficiently suppressed Tfh cells than CXCR5^+^ NK cells.

## Discussion

Growing evidence demonstrates that cellular immunology such as CD4^+^ and CD8^+^ T cells plays critical roles in the pathogenesis of MG [25]. However, the regulatory activity of NK cells, the innate immune component of cellular immune system, has been given less experimental attention. NK cells play important roles in the pathogenesis of several autoimmune diseases by modulating other components of the immune system. In this study, we investigated the roles and mechanism of NK cells in regulating EAMG by adoptive transferring splenic NK cells to recipient rats and by co-culturing NK cells or their subtypes with splenocytes or T cells in vitro.

NK cell treatment ameliorated EAMG symptoms, and this was associated with a reduction of anti-AChR97–116 IgG2a antibody levels and germinal center B cells. Previous study showed that depleting NK cell with anti-NK1.1 antibody decrease onset and severity of murine EAMG, which is contrary to our observations [26]. This discrepancy might attribute to the difference in interventions and animal models. NK1.1 are not exclusively expressed by NK cells, but also by some myeloid cells and T cells [27]. Thus, administration of anti-NK1.1 antibody might change these NK1.1-positive components, which make the results complicated to analyze.

Tfh cells are highly specified CD4^+^ T cell subset responsible for B cell proliferation, differentiation, and antibody production. In line with the decreased serum antibody levels and germinal center B cell percentages in NK-treated rats, our study indicated NK cell adoptive transfer reduced the percentages of Tfh cells. Further experiments confirmed these observations, as NK cells directly suppressed T cells and Tfh cells in vitro. Mechanically, NK cells could induce the apoptosis T cells and Tfh cells. Indeed, antigen-activated T lymphocytes express cell-surface NKG2D ligands and become susceptible to autologous NK cell lysis [28]. NK cells also kill the CD4^+^ T cell response through a TRAIL-dependent mechanism [11]. Previous studies demonstrated NK cells could also induce the apoptosis of neutrophil and eosinophil [29, 30]. Thus, cytotoxicity against other immune cells may serve as a general mechanism by which NK cells limit the inflammation and autoimmune response.

However, the usage of NK cell adoptive transfer for treating MG may be confined by the limited number of autologous NK cells which could be obtained. Although various culture methods have been developed to obtain sufficient number NK cell, ex vivo treatment may modulate the immunophenotypes and the subsequent functions of NK cells. A feasible alternative method is to activate or expand NK cells in vivo. IL-15 and its genetically modified super-agonist ALT-803, which has been used in clinical trials for treating tumors, can activate and expand NK cells and may be a promising candidate for treating MG. Indeed, IL-15 inhibited CD4^+^ T cells and Tfh cells in vitro. Similarly, IL-15 could increase apoptosis of CD4^+^ T cells. Additionally, IL-15 also inhibited LPS-induced B cell proliferation, and this might attribute to its inhibiting effects on T and Tfh cells. This was consistent with a previous study that NK cell could suppress the proliferation of B cells [31]. Thus, NK cells and their activators might be the new therapeutics methods in treating MG and other antibody-mediated autoimmune diseases in the future.

NK cells are the heterogenous lymphocytes with different phenotypes and functions. Different NK cell subtypes seems to play different even opposite roles in regulating immune response [32]. Our results indicated, according to the expression of CXCR5 molecular, NK cells could be divided into CXCR5^-^ and CXCR5^+^ NK subtypes. Phenotype analysis illustrated that, compared with CXCR5^-^ subset, CXCR5^+^ NK cells exhibited higher levels of ICOS and lower levels of CD27 and had a higher frequency of IL-17-producing cells. As CXCR5 is the chemokine receptor of CXCL13, which guides Tfh and B cells to migrate to B cell follicles, it is reasonable to assume that CXCR5^+^ NK cells could migrate to B cell area to modulate humoral response. One pioneer study showed that CXCR5^+^ NK could enter in lymph node follicles, and exert efficient control of viral replication within lymph nodes [33]. Our study indicated CXCR5^+^ NK cells could more efficiently migrate into B cell zone than the CXCR5^-^ subtype. More importantly, the frequencies of Tfh cells and germinal center B cells were positively correlated with CXCR5^+^ NK cell frequencies in EAMG rats. In addition, the frequencies of Tfh cells and germinal center B cells were negatively correlated with the ratios of CXCR5^-^ to CXCR5^+^ NK cells. These results indicated that CXCR5^-^ and CXCR5^+^ NK cells might have different roles in regulating Tfh cells or germinal center B cells. Ex vivo studies showed that CXCR5^-^ NK cells downregulated Tfh cells percentages more efficiently than CXCR5^+^ NK cells. Compared with CXCR5^-^ NK cells, CXCR5^+^ NK cells could enhance ICOS expression on Tfh cells. As ICOS is critical for antibody class switching [34], CXCR5^+^ NK cells might exhibit promotive effects on humoral immune response. As a whole, different NK cell subtypes exhibit different effects on humoral immune response and CXCR5^-^ NK cells but not CXCR5^+^ NK cells suppressed Tfh cells and inhibited the autoimmune response in EAMG models.

## Conclusions

Our results indicated NK cells ameliorated EAMG symptoms by suppressing Tfh cells and germinal center B cells, and this might attribute to the inhibitory role of CXCR5^-^ NK cells but not CXCR5^+^ NK cells. The function of CXCR5^+^ NK cells on Tfh and B cells need to be investigated in detail in the future, as the environments of *ex vivo* studies could not stringently simulate the micro-environments in vivo.

## Data Availability

The datasets used and/or analyzed during the current study are available from the corresponding author on reasonable request.

## Abbreviations

AChR: Acetylcholine receptor
BPM: Bovine peripheral myelin
CFSE: Carboxyfluorescein diacetate, succinimidyl ester
CXCR5: C-X-C chemokine receptor 5
DCs: Dendritic cells
EAE: Experimental autoimmune encephalomyelitis
EAMG: Experimental autoimmune myasthenia gravis
EAN: Experimental autoimmune neuritis
ICOS: Inducible co-stimulator
LPS: Lipopolysaccharide
MFI: Mean fluorescent intensity
MG: Myasthenia gravis
NK cells: Natural killer cells
PBS: Phosphate buffer saline
PD-1: Programmed death 1
PNA: Peanut agglutinin
Tfh cells: Follicular helper T cells
TRAIL: TNF-related apoptosis-inducing ligand
